# Evaluation of seed‐dispersal services by ants at a temperate pasture: Results of direct observations in an ant suppression experiment

**DOI:** 10.1002/ece3.10569

**Published:** 2023-09-29

**Authors:** Marie Konečná, Aleš Lisner, Petr Blažek, Pavel Pech, Jan Lepš

**Affiliations:** ^1^ Faculty of Science University of South Bohemia České Budějovice Czech Republic; ^2^ Research and Breeding Institute of Pomology Holovousy Czech Republic; ^3^ Biology Centre, Czech Academy of Science České Budějovice Czech Republic

**Keywords:** ant‐activity suppression, cafeteria experiment, myrmecochory, plant–ant mutualism, seed dispersal

## Abstract

Ants disperse seeds of many plant species adapted to myrmecochory. While advantages of this ant–plant mutualism for myrmecochorous plants (myrmecochores) have been previously studied in temperate region mostly in forests, our study system was a pasture. Moreover, we used a unique combination of observing the effect of ant‐activity suppression on ant dispersal and comparison of the contribution of ant and unassisted dispersal to the distance from mother plant. We established plots without and with ant‐activity suppression (enclosures). We offered diaspores of a myrmecochorous (*Knautia arvensis*), and a non‐myrmecochorous (*Plantago lanceolata*) species in a choice test and followed ants carrying diaspores during days and nights (focus of previous studies was on diurnal dispersal). We measured frequency and distances of ant dispersal and compared them with unassisted dispersal recorded using sticky trap method. The dispersal frequency was lower in enclosures (3.16 times). Ants strongly preferred diaspores of the myrmecochore to non‐myrmecochore with 586 and 42 dispersal events, respectively (out of 6400 diaspores of each species offered). Ant dispersal resulted in more even and on average longer distances (maximum almost tenfold longer, 994 cm) in comparison to unassisted dispersal. Ant dispersal altered the distribution of distances of the myrmecochore from roughly symmetric for unassisted dispersal to positively skewed. Ants dispersed heavier diaspores farther. Ants dropped the majority of diaspores during the dispersal (which reduces clustering of seeds), while several (11%) were carried into anthills. Anthills are disturbed microsites presumably favorable for germination in competitive habitats. Ants provided non‐negligible dispersal services to myrmecochorous *K. arvensis* but also, to a lesser extent, of non‐myrmecochorous *P. lanceolata*.

## Graphical abstract

We followed the journey of seeds of a myrmecochorous and non‐myrmecochorous species during diurnal and nocturnal ant dispersal. This unique approach with ant‐activity suppression and unassisted dispersal quantification allowed us to show the key role of ants in dispersal of myrmecochorous species and demonstrate non‐negligible contribution to non‐myrmecochorous species dispersal.

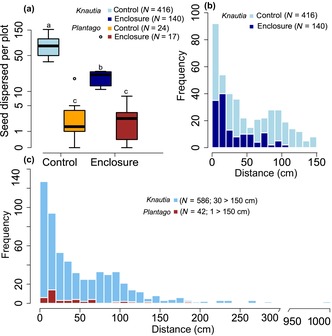

## 1 INTRODUCTION

Myrmecochory is a type of plant dispersal based on mutualism between ants and plants (Sernander, 1906). The ants are rewarded for seed dispersal of specialized plant species, that is, myrmecochores with an elaiosome, a nutritious appendage, particularly rich in amino acids in temperate conditions (Konečná, Moos, et al., 2018). An elaiosome is attached to a seed or fruit (together forming a diaspore; this term refers in further text to the dispersed unit; Sernander, 1906) and serves as food preferably for larvae (Fokuhl et al., 2012).

Elaiosomes are attractive to many ant species involved as mutualistic dispersers in myrmecochory (Christianini et al., 2012). However, ant species differ in the quality of their dispersal services and can be divided into two disperser guilds. Ants with larger bodies, solitary foraging and not feeding on elaiosomes in situ belong to high‐quality dispersers, in contrast to low‐quality dispersers with smaller bodies, recruit foraging and feeding on elaiosomes in situ (Hughes & Westoby, 1992; Leal et al., 2014; Wilker et al., 2022). Based on species‐specific perception (Bresinsky, 2014; Kjellsson, 1985), high‐quality dispersers are actively choosing among diaspores and thus not carrying the first diaspore they encounter (Hughes & Westoby, 1992; Leal et al., 2014).

The effectiveness of myrmecochory depends on multiple factors such as temperature and presence of other animals foraging for seeds (Ness & Bressmer, 2005). Another important factor is the identity of both ant and plant species. The attractiveness of diaspores for ants is a complex characteristic and may be influenced by multiple factors such as elaiosome metabolite composition (Reifenrath et al., 2012), elaiosome mass (Levine et al., 2019; Wendt et al., 2022), diaspore mass (Miller et al., 2020), and/or elaiosome:seed mass ratio (Leal et al., 2014; Levine et al., 2019). The diaspore mass is the most used generative trait (called seed weight) to express the reproductive effort as a part of three basic leaf‐height‐seed plant measures by Westoby (1998) and is, unlike the other mentioned traits, available in databases for wide range of species. While the relationship of seed mass and dispersal capacity is mostly studied indirectly by some traits, direct observations of dispersal distances in relationship with specific trait such as seed mass are less common (but see e.g., Skarpaas et al., 2011).

Plant species using exclusively myrmecochory for dispersal are very rare (Stamp & Lucas, 1983), since many plant species combine multiple consecutive dispersal agents (diplochory) with different advantages to optimize the dispersal output. Myrmecochorous species vary in investment in elaiosomes and thus diaspore attractiveness (Levine et al., 2019; Narbona et al., 2005) which is supposed to mirror the extent of their dependence on myrmecochory and other dispersal agents (but see Chen et al., 2019). Furthermore, ants can also carry seeds of non‐myrmecochorous species among other organic material (Barroso et al., 2013). Thus, the benefits of possessing an elaiosome should be adequate to the investment into the elaiosome. However, it was demonstrated that chemical signals might overpower the real benefit for ants (Turner & Frederickson, 2013). It is, therefore, important to quantify dispersal services by ants for myrmecochorous species in comparison with non‐myrmecochorous species.

In general, there are multiple mutually non‐exclusive benefits for plants connected with seed dispersal: *directed dispersal*, *colonization*, and *escape hypotheses* (Howe & Smallwood, 1982). Ants were documented for many species as the second dispersal agent after anemochory, endozoochory, and ballochory (Sernander, 1906; Vander Wall & Longland, 2004). The first stage of diplochory often results in escape from density‐dependent seed mortality (*escape hypothesis*) or even colonization of a new site, while the second agent might serve to reach a non‐random safe site for seedling recruitment, for example, better light conditions and/or nutrient‐rich substrate (*directed dispersal*; Dean et al., 1997; Oostermeijer, 1989; Vander Wall & Longland, 2004).

The importance of the three mentioned advantages of myrmecochory varies among different habitats, but even within habitat types, the results are sometimes contradictory (Giladi, 2006). In temperate open habitats, in which our focal locality belongs, *directed dispersal* hypothesis was supported by two out of six studies and *escape hypothesis* based on distance dispersal supported by 10 out of 13 (Giladi, 2006). The anthill or other species‐specific final location where the intact seed may be redispersed can serve as a safe site for germination (Ben‐Zvi et al., 2021; Gorb et al., 2000; Konečná et al., 2021). However, this is not always the case, since the seeds can be dropped on the way toward anthill (Gorb & Gorb, 1999). Moreover, <10% of seeds which arrive inside the anthill are not redispersed (Canner et al., 2012) and may stay too deep inside the nest to reach the surface and grow (Renard et al., 2010).

Previously, diurnal seed dispersal by ants in forests (or open habitats which were not grasslands) was mostly studied in temperate region without comparison with ant‐activity suppression. We added direct nocturnal observation of ant dispersal, since the ant night activity might be prominent. We studied ant dispersal services in a temperate pasture in an experiment with enclosures to suppress ant activity and intact controls. Enclosures to eliminate ant access inside plots have been rarely established in grasslands (but see Gibson, 1993; Turnbull & Culver, 1983). Additionally, we compared the contribution of ant dispersal relative to unassisted dispersal and to dispersal of a non‐myrmecochorous species.

This study aims to answer the following questions:

1. How does ant‐activity suppression influence the frequency and distance of diaspore dispersal?
2. How does the frequency and distance of ant dispersal differ between the myrmecochorous and non‐myrmecochorous plant species?
3. To what extent mutualistic ants increase the distance of seed dispersal compared to unassisted dispersal?
4. Is there a relationship between the distance of ant dispersal and seed mass?

## 2 MATERIALS AND METHODS

### 2.1 Study site

The study was conducted at a south‐facing grassland with slope of 8°. The study site is located in South Bohemia, Czechia, 49°4.87′N, 13°55.12′E, at 726 m a.s.l. The average yearly air temperature in 2016–2019 was 7.7 (derived from the meteodata measured at Husinec station, 6 km). Annual precipitation was 668 (station Zálezly, 3.5 km). During the dispersal experiment, average temperature was 19.2°C and precipitation 0.01 mm Vegetation belongs to the Violion caninae alliance with nutrient‐poor and moderately dry soils. Eight myrmecochores (Table 1 based on checklist of myrmecochores for Czechia; Chytrý et al., 2021; Konečná, Štech, & Lepš, 2018) and eight ant species (Table 2; ant nomenclature follows Seifert, 2018) with 74 aboveground anthills were found within the studied plots in 2016. The dominant ant species was non‐dispersing *Lasius flavus*, while the remaining species were dispersers. For more detailed description of the locality, see Konečná et al. (2021).

**TABLE 1:** Table of myrmecochorous plants at the locality based on the checklist of Czech Republic (Chytrý et al., 2021; Konečná, Štech, & Lepš, 2018).

Plant species	Family
*Arenaria serpyllifolia*	Caryophyllaceae
*Carex pilulifera*	Cyperaceae
*Knautia arvensis*	Dipsacaceae
*Luzula campestris*	Juncaceae
*Myosotis ramosissima*	Boraginaceae
*Potentilla verna*	Rosaceae
*Thymus pulegioides*	Lamiaceae
*Viola arvensis*	Violaceae

**TABLE 2:** Table with assigned the durability of nests and seed disperser status to each ant species present at locality (long‐lasting or seasonal), and quantification (proportion and number) of ant individuals carrying *Knautia* seeds.

Ant species	Durability of anthill	Seed disperser	Individuals observed carrying *Knautia* seeds	Number of observations
*Formica cunicularia*	Long‐lasting	Yes	Day (23.3%)	7
*Formica fusca*	Long‐lasting	Yes	Day (13.3%)	4
*Formica rufibarbis*	Long‐lasting	Yes		
*Lasius flavus*	Long‐lasting	No		
*Lasius psammophilus*	Seasonal and long‐lasting	Yes		
*Myrmica rubra*	Seasonal	Yes		
*Myrmica ruginodis*	Seasonal	Yes	Day (3.3%)	1
*Myrmica sabuleti*	Seasonal	Yes	Day (16.7%) and night (100%)	5 and 18
*Myrmica schencki*	Seasonal	Yes	Day (43.3%)	13
*Tetramorium* sp.	Long‐lasting	Yes		

*Note*: The frequency of dispersal by each species observed carrying *Knautia* seed during 1 day and 1 night.

### 2.2 Experimental design

**FIGURE 1:**
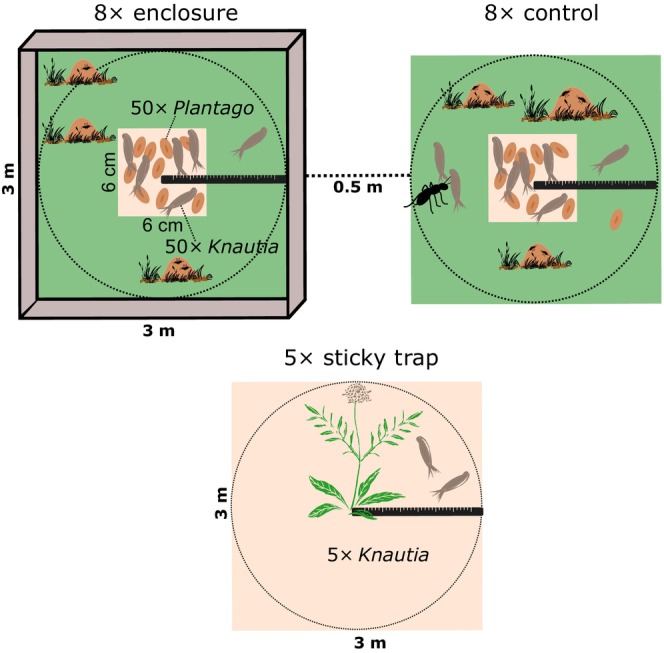
Scheme of the design of cafeteria experiment and sticky trap method.

Ant‐activity suppression (i.e., not complete ant exclusion which is not feasible in grasslands) was established in 2016 and consisted of 16 3 m × 3 m plots in a grid of 8 × 2 separated from neighboring plots by 0.5 m. Eight plots were intact controls and eight were enclosed to suppress ant activity in a checkerboard design (Figure 1 and Figure S1a). The light‐permeable plastic fence around the enclosures was 0.3 m high aboveground and 0.1 m deep in the soil. Additionally, insect glue was applied on the fence and ant‐specific poison (active substance Spinosad) was placed within the enclosures. Regular maintenance of the experiment included cutting grass around fences, reapplication of glue, and refilling poison traps.

### 2.3 Focal plant species

The resident species *Knautia arvensis* was chosen as the focal myrmecochore due to high attractiveness (in comparison with diaspores of other myrmecochores present at the locality in the pilot cafeteria experiment; Table 1) and sufficient availability of its mature diaspores at the time of the experiment. The species is adapted for dispersal by elaiosome, pappus, and hairs, thus this species is diplochorous, combining myrmecochory, anemochory, and epizoochory, while we speculate that the pappus is rather inefficient due to its relatively small size in comparison to seed (Figure S1b). We selected resident non‐myrmecochorous species *Plantago lanceolata* as a control. This species had the closest (yet lower) mean seed mass to *K. arvensis* (1.8 and 4.6 mg, respectively; Klotz et al., 2002) and we were able to gain its seeds in sufficient numbers from the commercial supplier (Planta Naturalis). The diaspores of *K. arvensis* were collected at several localities in South Bohemia (including the focal locality), then stored in a freezer to preserve the elaiosome attractiveness (Englický & Šerá, 2018; Prior et al., 2014).

### 2.4 Seed dispersal by ants

In cafeteria experiments, we compared the frequency and distance of seed dispersal between enclosures and controls and between the myrmecochorous and the non‐myrmecochorous species. The cafeteria experiment took place at eight observational days in two periods—4 days with favorable weather between July 1 and 7 and August 22–292,019. To enable the observation of dispersal in dense grassland vegetation, the plots were mown prior to the experiment. Each day, 50 diaspores of each species were exposed to ants on a bait station (cardboard squares 6 cm × 6 cm) placed in the center of each of the 16 plots. Diaspores of the two species were placed in a mixture in a single layer so ants were able to choose among them (between and within species) according to their attractiveness (Figure S1c).

After the start of the experiment, approximately noon, we repeatedly checked the position of seeds during dispersal and placed a marker where it was found (so the diaspore can be carried farther by the same or different ant). Diaspores were covered with fluorescent powder and approximately four people rotated among plots (time spent on plots as even as possible) and observed ants carrying diaspores during days and in ultraviolet light during nights (Figure S1c,d). The next morning the distances from the bait station of for both unique and ongoing dispersal events were measured, and all the present diaspores were collected (newly dispersed diaspores were also searched for and their distances measured). For additional details on methods of the direct observation of ant dispersal, see Text S1).

We distinguished two types of dispersal events: unique dispersal events with the diaspore present (i.e., found on the ground or seen to be carried into the anthill) and second type with diaspore absent at the last observation at the time of collection. These data we named ongoing dispersal events as one diaspore might be recorded repeatedly as more dispersal events during the dispersal process. The ongoing dispersal events mean that seeds were later dispersed to their final destination with a greater (often unknown) distance where they were either not found (e.g., inside the anthill) or found and counted as unique dispersal events, thus one diaspore can be counted multiple times in the ongoing dispersal events. Note that the number of the unique dispersal events is underestimated since some diaspores were taken into anthills and long‐distance dispersal events are harder to find. We consider the data on the unique events to be more reliable (each event represents one seed), and thus, the results in the main text are based on them. The data on the ongoing events, in which some diaspores may be counted as multiple dispersal events, are used for comparison and show similar pattern in Figure S2.

To see which ant species were acting as dispersers, we offered 10 diaspores of *K. arvensis* per plot for 1 day and night in August and collected the ants for identification.

### 2.5 Seed mass

All dispersed seeds were stored in the freezer after collection in the field until they were weighed. To examine the relationship of dispersal distance and seed mass of the myrmecochore, the mass of seeds (fresh state without drying) was measured including the remnants of fluorescent powder on their surface to determine the weight which the ants carried. Weighing of an elaiosome separately was limited by precision of analytical balances due to its low mass. Thus, we additionally weighed 16 replications of 10 elaiosomes and seeds without fluorescent powder to gain an average elaiosome:seed mass ratio. Seed mass of *P. lanceolata* was also measured in 16 replications of 10 seeds.

### 2.6 Unassisted dispersal of the myrmecochore

A sticky trap of the same size as the experimental plots of the cafeteria experiment (3 m × 3 m) was used to record unassisted dispersal (i.e., without any biotic dispersal agent, mostly by gravity and additionally wind). We used linoleum covered with insect glue to trap the falling diaspores (Figure S1e). Five full‐sized individuals (with majority of infructescences with mature seeds) of *K. arvensis* were fixed in a water container in the center of the plot for approximately 10 days. The procedure was repeated five times during July and August. The distances of trapped diaspores were measured in the same manner as in the case of ant dispersal, thus the dispersal by ants and unassisted dispersal can be compared.

### 2.7 Statistical analysis

The analyses were performed for both unique and ongoing dispersal event datasets. The analyses in the main text are based on unique dispersal events to avoid multiple observations of the same seed in the data of ongoing events, results of their respective analyses are in Figure S2.

To have comparable data from enclosures and controls, the dispersal events were restricted for all the analysis within a 150 cm radius circle. Frequencies of dispersal events are counted per plot out of a total of 400 diaspores offered. There was a gradient of ant activity (Figure S3) corresponding to a gradient of vegetation productivity across the locality (for details see Konečná et al., 2021). Productivity was decreasing while activity was increasing from first pair of plots, more in controls (quantified by number of dispersal events), thus eight columns (i.e., pairs of an enclosure and control) were used in all the analyses as a random factor. All the analyses were performed in R program (R Core Team, 2022; Wickham, 2016).

#### 2.7.1 Seed dispersal by ants

Frequency of dispersal events per plot or dispersal distances of individual dispersal events were used as response variables in separate generalized linear mixed‐effects models (GLMM). Both GLMM analyses were performed with plot type (i.e., enclosures and controls) and species as fixed‐effect factor (with the interaction) and the position on ant‐activity gradient (category 1–8) as a random factor in R package *glme* (Weerahandi et al., 2021). Poisson distribution was assumed for the frequencies and Gamma distribution for distances. Both analyses were performed with the log link function. Both GLMM analyses were followed by separate Tukey post hoc comparisons of dispersal frequency or distances between species and plot types in R package *emmeans* (Lenth et al., 2019).

#### 2.7.2 Seed mass

The relationship of dispersal distance gained by ants and seed mass was analyzed by linear regression after log_10_–log_10_ transformation.

#### 2.7.3 Unassisted dispersal of the myrmecochore

The log10‐transformed unassisted distances and distances gained in by ants were compared by Welch's *t*‐test.

## 3 RESULTS

### 3.1 Seed dispersal by ants

Out of 6400 seeds (8 days × 50 seeds × 16 plots) offered in total per species, we recorded 586 *K. arvensis* seeds and 42 *P. lanceolata* seeds dispersed from the bait station by ants. The ant species which acted as dispersers were mostly of genus *Formica* during the day and entirely of genus *Myrmica* during the night (Table 2).

**FIGURE 2:**
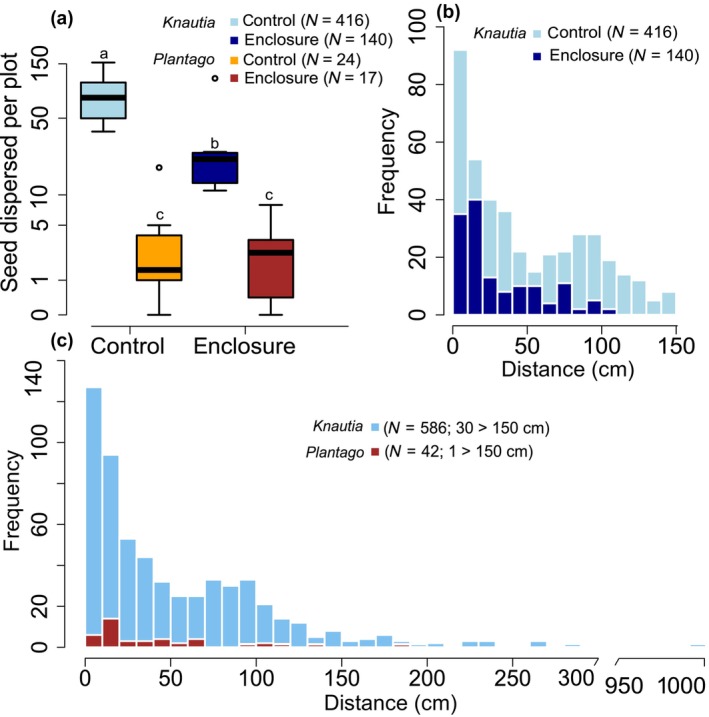
Ant‐dispersed seeds (a) boxplot of the frequency of dispersal events of *Knautia arvensis* and *Plantago lanceolata* in enclosures and controls per plot, letters signify the results of the Tukey test (b) histogram of distances of dispersal events of *K. arvensis* in enclosures and controls (c) histogram of distances of dispersal events of *K. arvensis* and *P. lanceolata* in controls including dispersal exceeding 150 cm (these values were excluded from statistical analyses to allow comparison of plot types, but are shown here to illustrate the tail of dispersal curve).

Ant activity was considerably suppressed in enclosures. This resulted in a lower count of dispersal events per plot in enclosures compared with the controls without activity suppression (*χ* ^2^ = 174.34, df = 1, *p* < .001; Figure 2a). Ants preferred the diaspores of *K. arvensis* to *P. lanceolata* (*χ* ^2^ = 275.33, df = 1, *p* < .001; Figure 2a). The response of the species in the frequency of dispersal events differed between plot types (i.e., significant species × plot type interaction; *χ* ^2^ = 4.28, df = 1, *p* = .038). The Tukey post hoc test showed differences in dispersal frequency between all the groups except within *P. lanceolata* species. Diaspores of *K. arvensis* were more frequently dispersed in controls than enclosures, while *P. lanceolata* was dispersed less and its diaspore dispersal frequency was not different between plot types (Figure 2a).

The dispersal distance did not significantly differ between *K. arvensis* and *P. lanceolata* (*χ* ^2^ = 1.30, df = 1, *p* = .253; median is 31.5 and 21 cm respectively; Figure 2c), while it differed between treatments (*χ* ^2^ = 37.38, df = 1, *p* < .001; Figure 2b). The distance of dispersal events significantly differed between species across the plot types (i.e., significant species × plot type interaction; *χ* ^2^ = 4.02, df = 1, *p* = .045). The Tukey post hoc test showed that diaspores of *K. arvensis* in controls were dispersed significantly farther than in enclosures.

When considering the dispersal events beyond the plots, (i.e., dispersal events >150 cm), the median is 35.3 and 22.5 cm for *K. arvensis* and *P. lanceolata*, respectively. Only one diaspore of *P. lanceolata* was dispersed outside the plots (185 cm) while there were 30 *K. arvensis* diaspores (Figure 2c). The maximum distance of a dispersal event was 994 cm (dispersal event by *Formica fusca*). In the sampling of ant individuals which were carrying diaspores, we discovered that the dispersal service for *K. arvensis* was provided by *Formica* and *Myrmica* during the day, whereas in the night exclusively by *Myrmica* (in our case *M*. *sabuleti*; Table 2).

The results of ongoing dispersal are in Figure S2. The Tukey test of frequencies showed the same pattern for both species and plot types as in case of unique dispersal events (i.e., ants preferred *K. arvensis* to *P. lanceolata* and while the dispersal of *K. arvensis* was more frequent in controls, the frequency of *P. lanceolata* dispersal was comparable in controls and enclosures).

### 3.2 Unassisted dispersal of the myrmecochore

**FIGURE 3:**
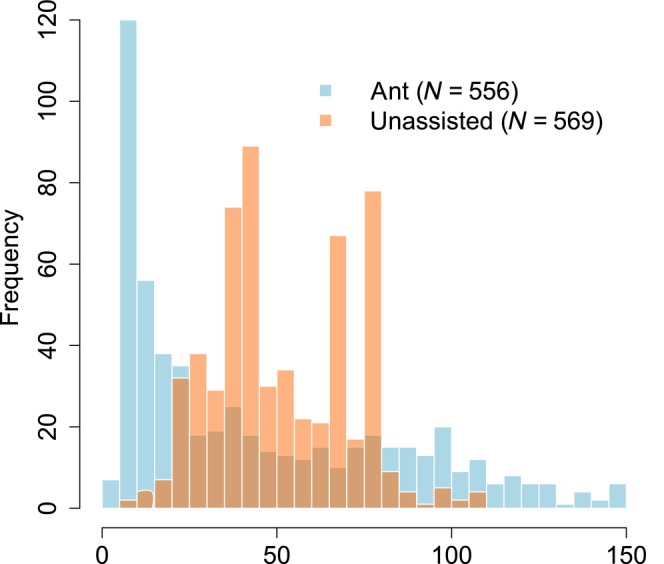
Histogram of all ant‐mediated dispersal events (<150 cm) and unassisted dispersal.

Across five replications (five plant individuals per each), there were 569 unassisted dispersal events in total with a maximum distance of 108 cm (median 46 cm; Figure 3 and Figure S4). While the median distances of both dispersal types were similar (ant dispersal within plots: median 32 cm), the maximum distance gained by ants (including beyond plots) was almost 10 times greater (994 cm). Thus, the relative contribution of ants to dispersal is considerable, with altered distribution of distances from symmetric for unassisted dispersal to positively skewed (i.e., with a long tail) for ant dispersal (Figure 3).

### 3.3 Seed mass

**FIGURE 4:**
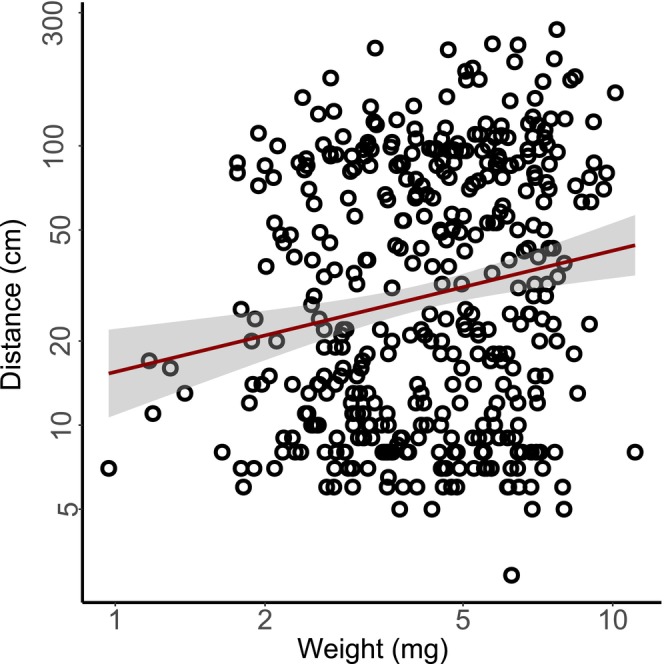
Linear regression of dispersal distance on diaspore weight of *Knautia arvensis* (in total 406 dispersal events with recovered diaspores) with 95% confidence interval (gray stripe).

The seeds of *K. arvensis* (including elaiosome) were heavier than *P. lanceolata* seeds (9.7 mg, SD = 1.3, and 1.4 mg, SD = 0.2, respectively). Heavier diaspores of *K. arvensis* were dispersed farther by ants (*F*_1,404_ = 13.6, *p* < .001; Figure 4). The elaiosome:seed mass ratio of *K. arvensis* was 0.082. The number of diaspores which could be weighed was 406, which is less than the number of unique dispersal events since some seeds were dispersed into the anthills and thus not recovered.

## 4 DISCUSSION

We quantified the dispersal frequency and distance of a myrmecochorous (*Knautia arvensis*) and a non‐myrmecochorous (*Plantago lanceolata*) plant species in plots with ant‐activity suppression and controls. We sufficiently suppressed the ant activity in enclosures, which resulted in lower diaspore dispersal frequency and distance of the myrmecochore. Mutualistic ants carried diaspores of the myrmecochore more frequently than non‐myrmecochorous species, and the difference was more pronounced in control plots. In addition, we experimentally assessed the relative contribution of the unassisted dispersal and thus demonstrated the importance of ants to dispersal of myrmecochores. Our findings indicate that dispersal services by ants are substantially increasing the dispersal distances for myrmecochores and can even contribute to the dispersal of non‐myrmecochorous species.

### 4.1 Ant activity

While feeding guilds of ants involved in myrmecochory are carnivores, omnivores, and fungivores (Christianini et al., 2012; Hughes et al., 1994), only omnivorous dispersers were present at our locality. Specifically, the dispersal service was provided by species from the genus *Myrmica* (exclusively seasonal anthills) and genus *Formica* (long‐lasting anthills). Ants from both genera were probably high‐quality dispersers sensu Hughes and Westoby (1992) and Leal et al. (2014) since they interacted with the seeds and chose them actively during our cafeteria experiment. Other characteristics of high‐quality dispersers are bigger body stature, not feeding on elaiosome in situ and solitary foraging. Bodies of workers of all present ants are much larger than the diaspores used. Foraging strategy is highly dependent on the size of collected food (Lanan, 2014), and there is no need for *Myrmica* or *Formica* to use group foraging to collect smaller loads. The maximum dispersal distance event observed in this study was performed by an individual of *Formica fusca* species. This is in line with the fact that the seed dispersal distances by Formicinae observed worldwide were much longer than by Myrmicinae (maximum 70 and 25.2 m respectively; Gómez & Espadaler, 2013).

To the best of our knowledge, night activity of ants as dispersers has not yet been considered. Night dispersal has barely been investigated in general (but see Weighill et al., 2017; dispersal by rodents), and the observations of myrmecochory are complicated as stressed by Bologna et al. (2017), but there are a few available methods for tracking dispersal by ants. We used fluorescent powder marking in this study (see also Chlumský et al., 2013), while others marked seeds with metal tags and tracked them by metal detector (Canner & Spence, 2011) or used video recording (Bologna et al., 2017). We observed diurnal dispersal with comparable frequency by *Myrmica* and *Formica*, while nocturnal dispersal mostly by *Myrmica*. Similarly, Źmihorski and Ślipiński (2016) observed more active individuals of the genus *Formica* during the day and *Myrmica* at night. The total ant activity (more dispersal events) in our experiment was greater during the night, probably due to heat avoidance (Oberg et al., 2012).

Few ant‐activity suppression experiments (see e.g., Klimeš et al., 2011; Wardle et al., 2011) and no complete elimination of ants have been conducted. At our locality, the anthill density (1 per 2 m^2^) was 250 times greater than in the forests (20 mounds/ha Wardle et al., 2011). Complete exclusion of ants is not possible at grasslands unlike in forests, but we substantially suppressed the ant activity, which resulted in decreased dispersal.

While there are many articles documenting no effect of fluorescent dye on ant behavior (e.g., Bestelmeyer & Wiens, 2003; García‐Meza & Martorell, 2022; Zelikova et al., 2008), it should be noted that the fluorescent powder might influence the ant behavior. We have not tested the effect of the powder, but as diaspores of both species were dyed, we assumed their dispersal was comparable.

### 4.2 Seed characteristics

Ants were choosing seeds according to their attractiveness within and between our target species, similar to Dostál (2005). During our cafeteria experiment, diaspores of the non‐myrmecochorous plant were also carried by ants (despite the lack of an elaiosome as a reward) which coincides with other studies (Barroso et al., 2013; Dostál, 2005). Seeds of non‐myrmecochorous species may even form a relatively big part of the ants diets and increase colony fitness of an omnivore (*Aphaenogaster senilis*; Barroso et al., 2013). However, ants preferred the diaspores of myrmecochorous to the non‐myrmecochorous species in our case. Besides the study by Dostál (2005) with similar results (which included some exceptions, e.g., myrmecochorous *Luzula campestris* was carried much less frequently than the non‐myrmecochorous *Dianthus deltoides*), this has hardly been studied experimentally.

Several diaspores of the myrmecochore were carried early in the morning, that is, a long time after the experiment started around noon of the previous day, despite that elaiosomes were supposedly dry and non‐attractive by that time. For example, Servigne and Detrain (2009) showed a 50% drop in seed attractiveness of *Viola odorata* after 7 h. We observed higher ant activity in the night resulting in many dispersal events even after 12 h since the start of the experiment. While this might be partly driven by activity of different ant species during days and nights, this pattern was probably mostly driven by the hot weather in summer which promotes night activity of ants and might be the cause of higher activity of *Myrmica* during nights (please note ant species activity was assessed based on small number of repetitions). Thus, ants probably did not discriminate diaspores of *K. arvensis* according to their freshness, unlike in previously studied species in temperate regions (Kjellsson, 1985; Servigne & Detrain, 2009). However, there might be a different seed trait (e.g., seed weight) based on which ants discriminate the diaspores, as they were inspecting the diaspores prior to the dispersal and actively choosing them within and between species. Diaspores of *K. arvensis* have a relatively low elaiosome:seed mass ratio 0.08 in comparison to other species such as *Chelidonium majus* 0.31, *Viola odorata* 0.18 (Servigne & Detrain, 2009) and relatively high seed mass in comparison to other non‐woody species (Šerá, 2004). Elaiosomes have higher weight loss caused by greater desiccation of elaiosomes relative to seeds due to their soft quality and higher water content (Servigne & Detrain, 2009). Heavier diaspores of the myrmecochore were carried farther by ants in our study (although the unexplained variation remained high). The opposite trend (i.e., negative relationship between seed mass and dispersal distance) was observed in a large number of species when accounted for the variability explained by plant height, while no significant relationship for ant‐dispersed plants was found when it was not accounted for height (Thomson et al., 2011). This could be explained by ant preference for higher quality diaspores with properly developed elaiosomes (and embryo; Ciccarelli et al., 2005). Another reason might be that longer dispersal events are performed by genus *Formica* with big individuals uninterested in smaller diaspores. We aimed to test the relationship of dispersal distance and germinability, but it failed due to a negligible germination rate. However, Vange et al. (2004) reported positive relationship of seed mass and germination rates for *K. arvensis*. Combination of this result with our finding that heavier diaspores are dispersed farther and thus are more attractive implies that the higher seed attractiveness for ants (and higher seed quality i.e., higher germination probability) might be causing longer dispersal distance. Another positive effect of ants is greater germination when elaiosome is removed (*Knautia dipsacifolia*; Mayer & Svoma, 1998).

### 4.3 Dispersal distance

As expected, diaspores of the non‐myrmecochorous species were carried to shorter distances than those of the myrmecochore in both controls and enclosures. Nevertheless, we showed in several cases non‐negligible dispersal distances of the diaspores without elaiosomes (one observation beyond the plots: 185 cm). The ant dispersal distance exceeded our expectations when designing the plot size (i.e., 150 cm radius) based on previous personal observations in temperate grasslands. We observed 30 dispersal events of the myrmecochore (5.1%) reaching beyond the plots (maximum almost 10 m). This distance, which we consider to be sufficient to avoid parent–offspring competition. Furthermore, myrmecochory can help to suppress intraspecific competition, since the diaspores of *K. arvensis*, a diplochorous species, were frequently dropped by ants during dispersal in our experiment. It corresponds with the results of Gorb and Gorb (1999), who observed this more often for diplochorous than exclusive myrmecochorous species. Thus, diaspores did not reach the anthill as the final destination of the dispersal in all cases.

The observed dispersal events were not sufficient to colonize new habitats in one step as proposed by Howe and Smallwood (1982), however, reaching a new suitable microsite within the locality by ants is rather effective and new locality can be reached gradually. Even though the long dispersal events are very rare (Nathan, 2006), they have a significant effect for meta‐population dynamics (Okubo, 1980).

While observed maximum dispersal distances for herbs using other dispersal types, especially anemochory, were huge (e.g., more than 4000 m for *Tussilago farfara*; Bakker, 1960), shorter distances were recorded for myrmecochory (median for mesophilous vegetation: 0.62 m; Gómez & Espadaler, 2013). Globally, 81% of recorded distances for myrmecochory were between 0 and 2 m, however, the dispersal curve has a long tail with maximum distances observed for sclerophyllous vegetation reaching 180 m (*Acacia ligulata*; *Iridomyrmex viridiaeneus*—Formicidae; median: 1.54 m; Whitney, 2002). The observed maximum for temperate regions and ant species present at our locality is 80 m (*Chelidonium majus*) and 15 m (various plant species, *Lasius niger*; Sernander, 1906), respectively. In temperate forest (*Melampyrum pratense*, with comparable seed mass 5.2 mg with our target species *K. arvensis*), the maximum dispersal distance was more than three times greater than of *K. arvensis* in our study (36.5 m; Chlumský et al., 2013). Nevertheless, the dispersal in dense grassland vegetation is probably more difficult than in forests with low understory cover. The dispersal efficiency of multiple steps in diplochory is not commonly studied (but see e.g., Chen et al., 2019). In our study, the average distance was very similar, while the distribution differed between positively skewed ant dispersal and symmetrically distributed unassisted dispersal. Moreover, for diaspores of the myrmecochore, longer dispersal events occurred, and seeds were also carried up the slope (unlike in unassisted dispersal), which is also important for more even distribution of diaspores.

Ants are distributing seeds with more even distances and with maximum dispersal distance almost 10× greater than unassisted dispersal. Distribution of ant dispersal was positively skewed in our study with high representation of short distances and occurrence of important longer dispersal events (more than 1.5 m), unlike unassisted dispersal. We acknowledge that rare unassisted dispersal events of longer distances may occur (e.g., in a windstorm), similar to the case of ant dispersal, and thus, both ant and unassisted dispersal may be underestimated. Furthermore, we would like to emphasize that frequent short distances reached by ant dispersal are missing in unassisted dispersal. However, ant dispersal should not lead to clustering since the natural starting position of the ant dispersal is not one spot as in our experiment but the distribution resulting from unassisted dispersal. Although we refer to unassisted dispersal, diaspores of *K. arvensis* are also potentially wind dispersed. However, the observed dispersal was rather ineffective in terms of distance, being rarely longer than plant height. The species is also able to disperse by epizoochory (bristles and toothed calyx), but the retention time on the dispersing agent is very short in comparison with other species (500 m were rarely reached in the experiment with a fox dummy (Hovstad et al., 2009). Nevertheless, while providing long distances, neither anemochory nor epizoochory can precisely target a suitable microsite, ants might provide directed dispersal as the last dispersal agent of diplochorous species (Vander Wall & Longland, 2004).

The final dispersal distance gained by the seed of a diplochore from the parent plants consists of more dispersal phases combined. It is, however, not a simple sum of multiple subsequent dispersal events of the diplochorous species since each dispersal phase may have a different direction. The bait station could be considered as a cluster of seeds from fallen infructescence, but realistically the seeds are likely to be scattered and thus harder to find for ants. The dispersal distance is also assumed to depend on plant height, that is, the position of the infructescence (Thomson et al., 2011).

### 4.4 Directed dispersal

Supporting the directed dispersal (i.e., precise targeting on a favorable microsite), we observed several diaspores directly carried inside the anthills, which is not always the final destination of the dispersal (Canner et al., 2012; Gómez & Espadaler, 1998). Kjellsson (1985) reported around 80% of *Carex pillulifera s*eeds which were not dispersed to the final location where they may germinate (combination of *Formica*, *Leptothorax* and *Myrmica*). The final spot for the seed after using an elaiosome is a species‐specific refuse pile inside or outside the anthill, on the territory border, or haphazardly within the territory (Gómez & Espadaler, 1998). The optimal option for directed dispersal are dispersers with anthills lasting a few years (in our case *Formica* species), that is, forming disturbances of intermediate durability (sensu Huston, 1979). These ants provide dispersal services targeted to the anthills favorable for germination and maintaining diversity (the same locality; Konečná et al., 2021) and moreover dispersing for longer distances than *Myrmica* ants with seasonal nests. In this study, anthills were reached only in 11% of observed dispersal events. On the other hand, the dominant ant species (*Lasius flavus*) creates in our study system long‐lasting anthills but does not disperse seeds, thus, these favorable disturbed microsites are not reachable by ant dispersal.

### 4.5 Escape hypothesis

The observed dispersal was sufficient for the diaspores to escape from the negative effects of the parent plant in their vicinity. These negative effects are predators, pathogens (Manzaneda et al., 2005), and intraspecific competition (Canner et al., 2012; Prior et al., 2014). Ants may save several seeds from predation when they disperse them before a predator finds them (Heithaus, 1981). This was observed in our experiment where several seeds were carried into anthills. This may provide the escape, for example, from earwigs, which we observed in August (in contrast to July) to prey on seeds in the night. Moreover, the presence of ants leads to spatial avoidance of some seed predators (e.g., carabids; Hawes et al., 2013). We speculate that the intraspecific competition is relatively less important in productive meadows than the effect of predators and pathogens when the final destination of dispersal is not an anthill. The reason is comparable interspecific competition at the final location of dispersal to the intraspecific competition near the parent plant. We can speculate that the intraspecific competition between plants was suppressed enough by ants since the unassisted dispersal resulted in more clustered diaspores than when dispersed by ants.

A myrmecochorous species was preferred to the non‐myrmecochorous one in our cafeteria experiment. We directly observed ants carrying seeds into anthills in 66 cases out of 556 dispersal events. Ants with additional 64 diaspores were clearly heading toward anthill. These dispersal events support the directed dispersal hypothesis. However, anthills as presumably favorable microsites are not always reached by ant dispersal, since seeds are dropped earlier. This is not always disadvantageous, since it reduces intraspecific competition as it prevents clustering of seeds (and seedlings in turn) either under the mother plant or at an anthill (support of escape hypothesis). The observed dispersal distances were not sufficient to observe possible colonization of a new habitat. The distance gained by ants was longer and spatially more evenly distributed (including dispersal up the slope) than by unassisted dispersal. Ants at temperate pastures may provide either safe sites for seedling germination (however seeds must come from seed rain in the case of *Lasius flavus* and long‐lasting anthills), dispersal services (*Myrmica* species forming seasonal anthill) or a combination of both (*Formica* species).

## AUTHOR CONTRIBUTIONS

**Marie Konečná:** Conceptualization (equal); data curation (lead); formal analysis (lead); investigation (equal); methodology (lead); validation (lead); visualization (lead); writing – original draft (lead); writing – review and editing (equal). **Aleš Lisner:** Formal analysis (supporting); investigation (equal); methodology (supporting); validation (supporting); visualization (supporting); writing – review and editing (equal). **Petr Blažek:** Formal analysis (supporting); investigation (equal); methodology (supporting); validation (supporting); visualization (supporting); writing – review and editing (equal). **Pavel Pech:** Conceptualization (supporting); investigation (supporting); methodology (equal); writing – review and editing (equal). **Jan Lepš:** Conceptualization (equal); formal analysis (supporting); funding acquisition (lead); methodology (equal); supervision (lead); validation (supporting); visualization (supporting); writing – original draft (supporting); writing – review and editing (equal).

## FUNDING INFORMATION

The research was supported by the Czech Science Foundation—GACR 23‐05654S.

## CONFLICT OF INTEREST STATEMENT

None.

### OPEN RESEARCH BADGES

This article has earned Open Data, Open Materials and Preregistered Research Design badges. Data, materials and the preregistered design and analysis plan are available at https://doi.org/10.5061/dryad.s4mw6m9cf.

## Supporting information


Appendix S1

Data S1

## Data Availability

Data are available in the Dryad Digital Repository: https://doi.org/10.5061/dryad.s4mw6m9cf and supporting information (Data S1).
