# Supplementary material for: Evaluation of seed‐dispersal services by ants at a temperate pasture: Results of direct observations in an ant suppression experiment
Source: Ecol Evol. 2023 Sep 29;13(10):e10569. doi: 10.1002/ece3.10569 (PMC10541265; doi:10.1002/ece3.10569)
Supplement: Supplementary file 1 — Appendix S1 [file ECE3-13-e10569-s001.pdf]

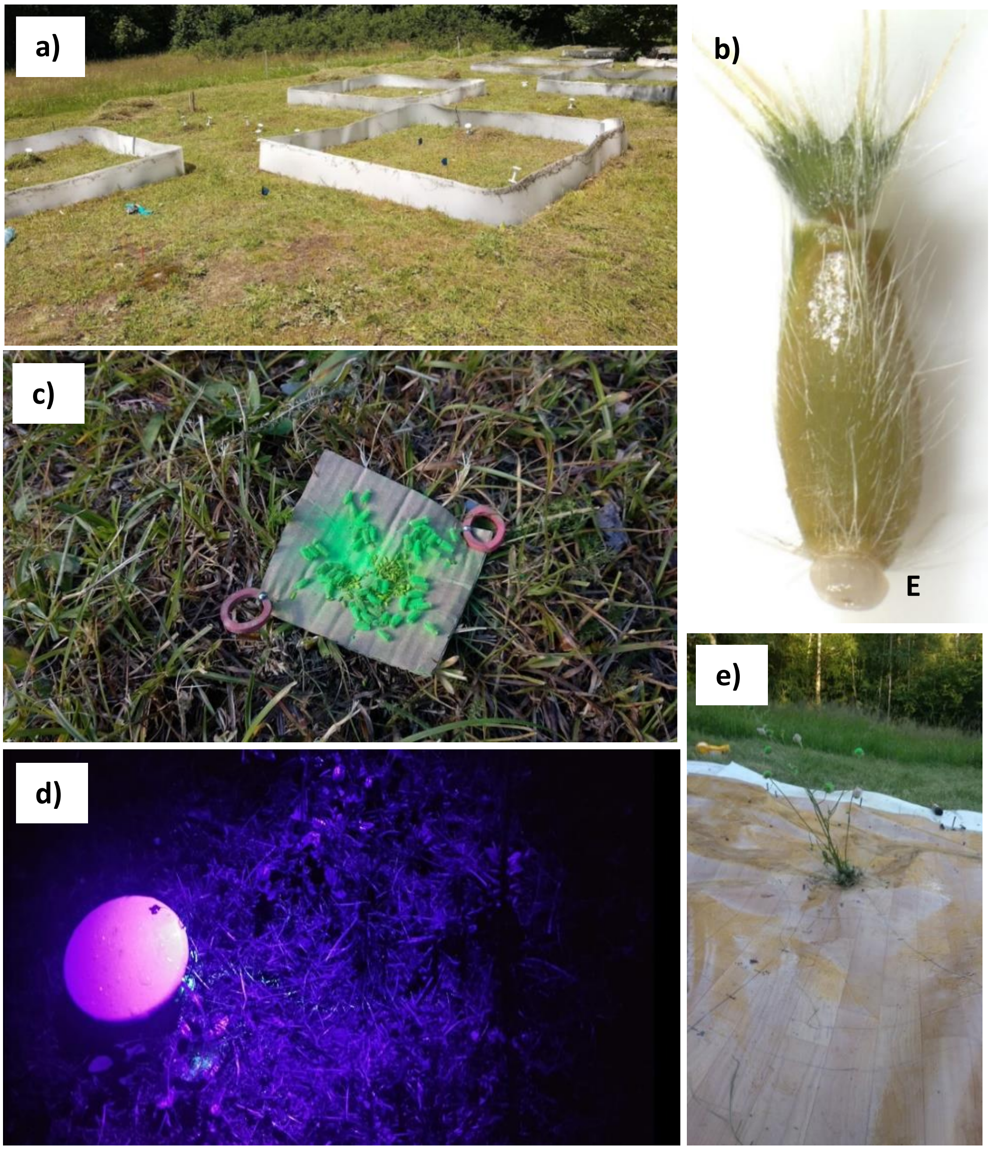

Figure S1 Photos of experiment a) Ant enclosures and control plots in checkerboard design, b) seed of *Knautia arvensis* with elaiosome (E), c) placement of seeds in the centre of plot at a bait station in the cafeteria experiment d) trail of fluorescent powder after seed dispersal in UV light and e) the sticky trap with five plants *Knautia arvensis* in the centre.

Text S1: detailed description of ant dispersal observation

The start of the cafeteria experiment with direct observation of ant dispersal combined with visual diaspore search (during the night with UV light) was at around 10 a.m. Then the plots were repeatedly investigated for diaspores during dispersal or ants interacting with the diaspores on the bait station. When no activity was observed, the observer continued to the next plot (time spent on plots as evenly as possible).

We observed the ants carrying diaspores three times in the night with the last observation at 3 a.m. At this time, all dispersed diaspores were given into Eppendorf tubes and placed back next to the marker on the final spot of dispersal. The next morning, all the recovered diaspores were collected including the non-dispersed diaspores on the bait station. When we observed some diaspores being dispersed or already dispersed during the diaspore collection, we also included them among dispersal events. The last observation was performed the next morning between 8 – 9 a.m. when we were collecting the marked diaspores and measuring the distances. The observations were done on consecutive days when the weather allowed. However, the last morning observation was done following each observation day regardless the weather.

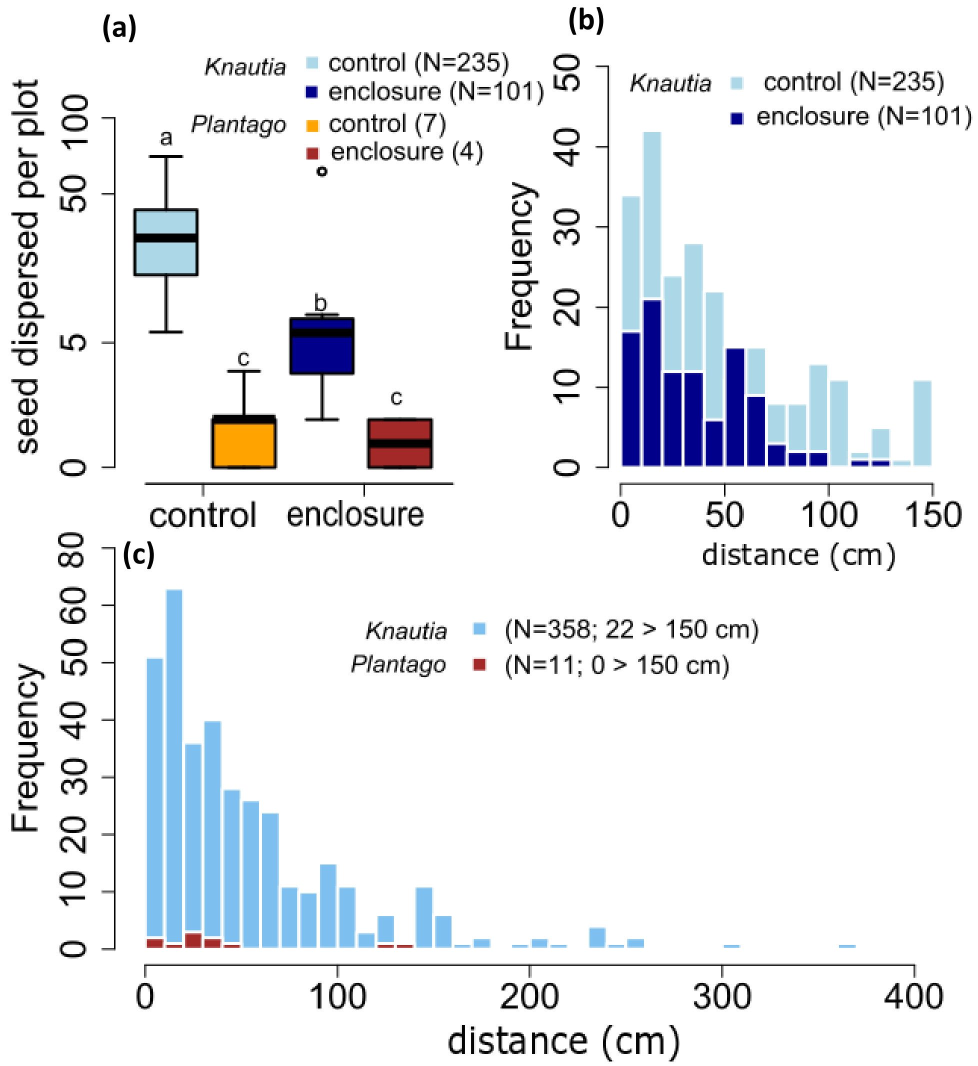

Figure S2 Results of ongoing dispersal events a) boxplot of the frequency of dispersal events of *Knautia* and *Plantago* in enclosures and controls per plot, letters signify the results of the Tukey test b) histogram of distances of dispersal events of *Knautia* in enclosures and controls c) histogram of distances of dispersal events of *Knautia* and *Plantago*. In total, we observed 358 measurements of ongoing dispersal events (i.e. seed was not present at the time of observation) of *Knautia* (22 distances > 150 cm; maximum: 369 cm, median: 40.5 cm) and 11 for *Plantago* (no distances > 150 cm; maximum: 137 cm, median: 26 cm).

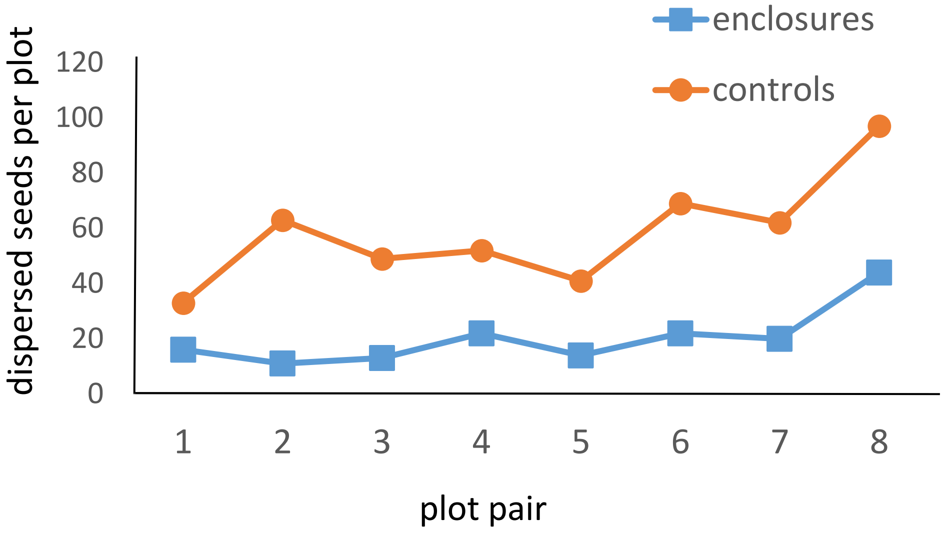

Fig S3 Seeds dispersed per a plot (1-8 is the position of plot pair enclosure and control at a productivity gradient, 1 is highest productivity and 8 lowest). The activity is increasing more in controls than in enclosures.

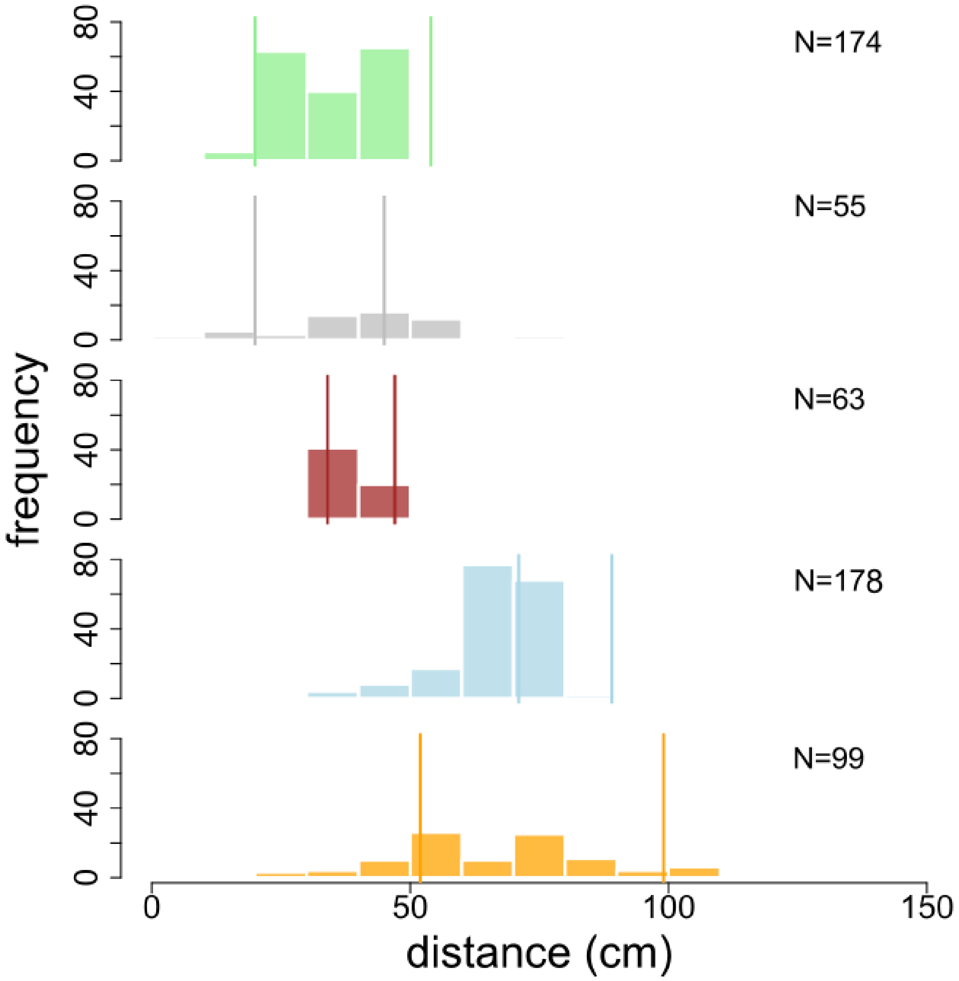

Figure S4 Results of five consecutive separate repetitions of unassisted dispersal of *Knautia arvensis* Each histogram is result of five individuals and the vertical lines indicate the position of the lowest and highest infructescence.
